# Oncolytic virotherapy reverses the immunosuppressive tumor microenvironment and its potential in combination with immunotherapy

**DOI:** 10.1186/s12935-021-01972-2

**Published:** 2021-05-13

**Authors:** Yalei Zhang, Ye Li, Kun Chen, Ling Qian, Peng Wang

**Affiliations:** 1 Department of Integrative Oncology, Fudan University Shanghai Cancer Center, 270 Dong An Road, Shanghai, 200032 China; 2 Department of Oncology, Shanghai Medical College, Fudan University, Shanghai, 200032 China

**Keywords:** Tumor microenvironment, Oncolytic virus, Immunotherapy, Combination therapy

## Abstract

It has been intensively reported that the immunosuppressive tumor microenvironment (TME) results in tumor resistance to immunotherapy, especially immune checkpoint blockade and chimeric T cell antigen therapy. As an emerging therapeutic agent, oncolytic viruses (OVs) can specifically kill malignant cells and modify immune and non-immune TME components through their intrinsic properties or genetically incorporated with TME regulators. Strategies of manipulating OVs against the immunosuppressive TME include serving as a cancer vaccine, expressing proinflammatory factors and immune checkpoint inhibitors, and regulating nonimmune stromal constituents. In this review, we summarized the mechanisms and applications of OVs against the immunosuppressive TME, and strategies of OVs in combination with immunotherapy. We also introduced future directions to achieve efficient clinical translation including optimization of preclinical models that simulate the human TME and achieving systemic delivery of OVs.

## Background

Increasing studies have been focused on the role of tumor microenvironment (TME) in immunosuppression. Hypoxia, acidosis, low immunogenicity and suppressed immune cells in the TME pose a great challenge to cancer immunotherapy [1]. Although tremendous progress has been achieved in immune checkpoint blockade (ICB) and chimeric antigen T (CAR-T) cell therapies, considering the heterogeneity and immunosuppression of the TME in many tumors, these two leading immunotherapies that require a pre-existing inflammatory microenvironment for optimal efficacy are not a panacea. The limited response rate in ICB-treated patients and modest efficacy of CAR-T cell therapy for solid tumors, especially for those tumors with immunosuppressive TME remain as intractable problems. The current predicament of immunotherapy raises an imperious demand for a proinflammatory shift of the TME [2, 3].

Oncolytic viruses (OVs) are a type of replicative-competent agents that selectively infect and lyse tumor cells and reverse immunosuppression by targeting the TME including both immune and non-immune stromal constituents [4]. As a versatile therapeutic agent, an OV can intrinsically trigger tumor-specific immune responses or be genetically inserted with exogenous therapeutic genes to modulate the TME, bringing potent therapeutic efficacy and relatively low toxicity [5].

In this review, we summarized the modulatory effects of OVs against the immunosuppressive TME as well as the preclinical and clinical applications of OVs in combination with immunotherapy. Developing humanized animal model to simulate human TME and optimizing administration methods of OVs were also discussed.

## Immunosuppression in the TME

**Fig. 1:**
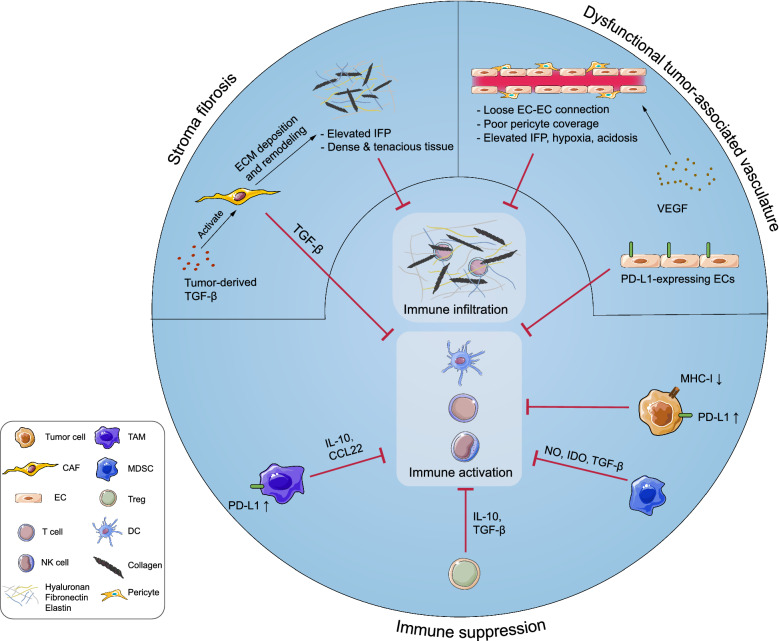
Components of tumor microenvironment (TME) contribute to the immunosuppression in various manners. Tumor cells downregulate expression of major histocompatibility complex-I (MHC-I) and antigens to avoid antigen presentation and T cell recognition, and express immune checkpoint proteins such as programmed cell-death ligand 1 (PD-L1) and cytotoxic T lymphocyte-associated antigen 4 (CTLA-4) to inactivate infiltrated T cells. Additionally, tumor cells recruit various immunosuppressive cells [e.g. myeloid-derived suppressor cells (MDSCs), tumor-associated macrophages (TAMs), and regulatory-T cells (Tregs)] by expressing immunosuppressive molecules [e.g. interleukin (IL)-10, chemokine ligand (CCL)-5, granulocyte–macrophage colony-stimulating factor (GM-CSF), indoleamine-2,3-dioxygenase (IDO) and tumor growth factor-β (TGF-β)]. Tumor cells, immunosuppressive cells and various immunoregulatory molecules [e.g. reactive oxygen species (ROS), arginase-1 (Arg-1), CCL-22, IL-10, and PD-L1] construct an immunosuppressive network in the TME. The activities of dendritic cells (DCs), T cells, natural killer (NK) cells, and other immune cells are therefore repressed severely. Moreover, classical stromal components contribute to immunosuppression. Continuous release of tumor-derived vascular endothelial growth factor (VEGF) leads to the formation of dysfunctional blood vessels with loose endothelial cell (EC)-EC connections and poor pericyte coverage, which exacerbates hypoxia and acidosis in the TME, thereby impairing the functionality of immune cells. Activated cancer associated fibroblasts (CAFs) lead to excessive extracellular matrix (ECM) deposition, which results in dense and tenacious fibrotic tissue surrounding the tumor mass and an elevated interstitial fluid pressure (IFP). These formidable physical barriers severely hinder immune infiltration and drug perfusion

The TME consists of cellular and non-cellular components. The cellular components include neoplastic cells, cancer-associated fibroblasts (CAFs), endothelial cells (ECs), innate immune cells [e.g., neutrophils, dendritic cells (DCs), and natural killer (NK) cells], adaptive immune cells (e.g., T and B cells), and immunosuppressive cells [e.g. myeloid-derived suppressor cells (MDSCs), tumor-associated macrophages (TAMs), and regulatory T cells (Tregs)]. The non-cellular components include the extracellular matrix (ECM), tumor vasculature, and secretory molecules (e.g. cytokines, chemokines, growth factors, and proteases) [6]. Notably, the majority of TME components contribute to the immunosuppressive microenvironment in various manners, as is shown in Fig. 1.

### Tumor cells and tumor-associated immune cells construct an immunosuppressive network

The sophisticated interactions of tumor cells, tumor stroma, and the host immune system construct a highly immunosuppressive TME as a tumor develops. Malignant tumor cells escape from host immunosurveillance by various mechanisms that include downregulating major histocompatibility complex (MHC)-I to escape T cell recognition, expressing immunoinhibitory surface proteins such as programmed cell death protein ligand-1 (PD-L1) to inactivate cytotoxic T cells (CTLs), and secreting immunosuppressive cytokines [e.g. transforming growth factor-β (TGF-β), granulocyte–macrophage colony-stimulating factor (GM-CSF), and interleukin (IL)-10], chemokines [e.g. chemoattractant cytokine ligand (CCL) 20 and CCL17], indoleamine-2,3-dioxygenase, and other secretory factors, thereby inhibiting T cell proliferation, inducing recruitment of immunosuppressive cells such as Tregs and MDSCs, and promoting phenotype conversion of macrophages from anti-tumor M1 to pro-tumor M2-like TAMs [7, 8]. Recruited immunosuppressive cells impair host immunosurveillance through various mechanisms. TAMs promote immunosuppression by producing IL-10 and TGF-β, and recruit Tregs by producing CCL22 [9, 10]. In terms of MDSCs, arginase-1 expressed by MDSCs degrades L-arginine required for T cell proliferation, and T cell receptor signaling is downregulated by MDSCs, which contributes further to T cell inactivation. MDSCs also recruit Tregs by secreting chemokines such as CCL3, CCL4, and CCL5 [11]. Tregs exert a wide range of immunosuppressive effects by secreting immunosuppressive cytokines or acting in a contact-dependent manner, such as inhibiting antigen presentation of DCs, halting early expansion of T cells, downregulating proinflammatory IL-12 signaling by expressing a competitive receptor [12], repressing the expression of T cell-associated cytokines (e.g. IFN-γ, TNF-α, and IL-2), and inhibiting B cells, NK cells, and other immune cells [13, 14]. Collectively, densely distributed negative immune cells and anergic T cells have been recognized as common features of the immunosuppressive TME, which indicate a poor prognosis [15].

### Non-immune stromal components and immunosuppression

Tumor-associated vasculature contributes to immunosuppression to a great extent. Rapid progression of a tumor requires tremendous amounts of oxygen and nutrients, which renders the TME relatively hypoxic, resulting in excessive production of proangiogenic factors such as vascular endothelial growth factor (VEGF) during tumorigenesis, this leads to abnormal tumor vasculature with loose EC-EC connections, poor pericyte coverage, a leaky structure, and chaotic organization. Such vasculature with poor delivery functions further exacerbates hypoxia and acidosis as well as elevates interstitial fluid pressure (IFP) of the TME, which create an unfavorable condition for T cell proliferation, infiltration and activation [16]. Apart from contributing to disrupted vasculature, many proangiogenic factors also possess immunosuppressive properties. As an example, VEGF has direct immunosuppressive effects by recruiting immunoinhibitory cells to suppress the activity of T cells and disrupting the maturation and antigen-presenting ability of DCs [17, 18]. Tumor-associated ECs also contribute to T cell inactivation by expressing PD-L1 [19].

In addition to tumor vasculature, other stroma components also contribute to immunosuppression. Activated by tumor-derived molecules such as TGF-β and IL-6, CAFs induce excessive deposition of ECM proteins (mainly collagen) and create a dense and tenacious stroma with elevated IFP, which forms a formidable physical barrier for intratumoral infiltration of immune cells. Additionally, CAF-derived immunosuppressive molecules, such as TGF-β, CXCL2, CCL5, IL-6, and IL-10, contribute to immunosuppression by recruiting immunosuppressive cells such as MDSCs and maintaining their suppressive phenotype. Prevention of T cell infiltration, induction of T cell apoptosis, and impaired cytotoxicity of both NK and T cells have been shown to be mediated by CAFs [20, 21].

### Role of the immunosuppressive TME in outcomes of immunotherapy

After the discovery and clinical introduction of ICB and CAR-T cell therapies, there has been a surge in the development of deleveraging the immune system to treat cancer [22–25]. In ICB therapy, leading checkpoint inhibitors, including anti-cytotoxic T lymphocyte-associated antigen 4 (CTLA-4) and anti-programmed cell-death protein 1(PD-1)/PD-ligand (PD-L1) monoclonal antibodies, have reached promising clinical outcomes. Ipilimumab, an anti-CTLA4 antibody, was approved for treatment of melanoma in 2011 [26]. Anti-PD-1/PD-L1 antibodies, such as pembrolizumab, nivolumab, and atezolizumab, have also progressed to clinical applications for various tumors such as non-small cell lung cancer (NSCLC), head and neck cancer, and colorectal cancer [27–29]. CAR-T cell therapy is another promising immunotherapy that modifies T cells to be specifically redirected to TAAs on tumor cells. CTL019, an anti-CD19 CAR-T cell product, was approved by the FDA for treatment of refractory B-cell lymphoma [30].

Although desirable therapeutic outcomes have been yielded in some patients, insufficient efficacy and limited responses have been reported for both ICB and CAR-T cell therapies in patients with an immunosuppressive TME phenotype [31]. ICB and CAR-T cell therapies exert anti-tumor effects mainly in a CTL-dependent manner. Preclinical and clinical studies have revealed that the therapeutic efficacy of targeting CTLA4 and PD-1/PD-L1 is closely associated with high infiltration of PD-1 expressing CD8 + T cells, expression of T-helper type 1 genes, and expression of PD-L1 and CTLA4 within tumors at baseline [32–36]. However, the functionality and intratumoral infiltration of CTLs are severely hindered by immunosuppressive and non-immune stromal components that create a non-T cell inflamed TME, characterized by an absence of tumor antigen presentation and sequestered CD8 + T cells around the tumor margin with low expression of cytotoxic markers, such as IFN-γ and granzyme B[37]. This immunogenically “cold” TME is seen in many patients with hypo-immunogenic tumors such as colorectal carcinoma (CRC) and pancreatic ductal adenocarcinoma (PDAC) [37, 38]. Therefore, a rational therapeutic strategy is to introduce a TME-reprogramming agent into immunotherapy. As a versatile genetic engineering platform to reshape the TME, OVs have emerged as a leading candidate to facilitate anti-tumor immunotherapy.

## Current development of oncolytic virotherapy

OVs are a class of biological agents with tumor selectivity and replication competence. In the nineteenth century, tumor regression was observed after viral infection, which introduced virotherapy as a therapeutic option to treat cancers [39]. In 1991, the first genetically engineered OV, a herpes simplex virus 1 (HSV-1) mutant with deficient thymidine kinase, was created for treatment of malignant glioma in nude mice [39]. After OV-induced anti-tumor immune activation was revealed by pre-clinical and clinical studies in recent years, oncolytic immunotherapy opened up a new chapter in anti-tumor therapy [40, 41].

OVs are classified into naturally occurring viruses and genetically engineered viruses. The majority of OVs are genetically modified to achieve tumor selectivity and reduce toxicity [5]. Take H101 (Oncorine), an adenovirus recombinant as an example, the coding region responsible for E1B55K protein to inactivate p53 for viral replication in normal cells was deleted, leaving this virus only replicable in malignant cells with aberrant p53 function [42]. In addition, they serve as a powerful transgene-delivering tool by genetically modified with various regulatory molecules to fight against the immunosuppressive TME, such as inserting immunostimulatory factors to potentiate anti-tumor immunity, or ECM-modifying agents to enhance intra-tumoral infiltration of immune cells[5]. The mechanisms and applications of OVs against the TME will be introduced later. To date, a great number of OV agents originate from different species of OVs have been applied in clinical investigations. The biological properties and genetic modifications of the major types of OVs, and clinical applications of some leading OV agents were summarized, as is shown in Table 1.

**Table 1:** Leading OV candidates applied in clinical trials

OV type	Genome structure	OV mutant	Genetic modification	Clinical application	Administration approach
HSV-1	Double stranded DNA	T-Vec	ICP34.5 and ICP47 deletion GM-CSF insertion	Melanoma, sarcoma, head and neck cancer, breast cancer, colorectal cancer, pancreatic cancer	Intratumoral
		HF10	UL43, UL49.5, UL55, UL56, and LAT deletion	Melanoma, breast cancer, pancreatic cancer	Intratumoral
		HSV1716	ICP34.5 mutation	Late-stage pediatric cancers, melanoma, hepatocellular carcinoma, glioblastoma,mesothelioma, neuroblastoma	Intratumoral, intravenous
Adenovirus	Double stranded DNA	ONYX-15	E1B55K deletion	Pancreatic cancer, colorectal cancer, head and neck cancer, ovarian cancer	Intratumoral
		H101	E1B55K & partial E3 deletion,	Head and neck cancer	Intratumoral
		LOAd703	E1ACR2 deletion, E2F-binding sites insertion TMZ-CD40L & 4-1BBL insertion	Pancreatic cancer, melanoma	Intratumoral
		VCN-01	E1ACR2 deletion, E2F-binding sites insertion, PH20 hyaluronidase insertion, RGD insertion in the fibre knob	Head and neck cancer, retinoblastoma, pancreatic cancer	Intratumoral, intravenous, intravitrous
		Telomelysin (OBP-301)	hTERT insertion	Solid tumors	Intratumoral
		ONCOS-102	GM-CSF insertion	Peritoneal malignancies, prostate cancer	Intratumoral
Vaccinia virus	Double stranded DNA	Pexa-Vec (JX-594)	thymidine kinase mutation GM-CSF, lacZ insertion	Hepatocellular carcinoma, colorectal cancer, solid tumors	Intratumoral, intravenous
		GL-ONC1	Ruc-GFP, lacZ, gusA insertion	Head and neck cancer, ovarian cancer	Intravenous
Parvovirus	Single stranded DNA	H-1PV (ParvOryx)	/	Glioblastoma, pancreatic cancer	Intratumoral, intravenous
Reovirus	Double stranded RNA	Reolysin® (pelareorep)	/	Melanoma, breast cancer, prostate cancer, ovarian cancer, multiple myeloma, pancreatic cancer, colorectal cancer, non-small cell lung carcinoma	Intravenous
Measles virus	Single stranded RNA	MV-NIS	Sodium-iodide symporter insertion	Multiple myeloma	Intravenous
Coxsackie virus	Single stranded RNA	CAVATAK	/	Melanoma, breast cancer, prostate cancer, bladder cancer, non-small cell lung carcinoma	Intratumoral

### Herpes simplex virus 1 (HSV-1)

HSV-1 is an enveloped, double-stranded linear DNA virus with a large genome of 152 kb. Owing to the large genome size, HSV allows incorporation of large exogenous DNA, conferring a more versatile transgene capability to HSV. The robust virulence and immunogenicity of HSV-1 provide a “double-edge sword” effect. Potent virulence and immunogenicity enable efficient oncolysis and anti-tumor immune responses. However, rapid immune-mediated viral clearance and potential cytotoxicity result in delivery and safety issues [40].

To achieve tumor selectivity and optimized efficacy, several HSV mutants have been created and applied to many types of tumors. As the first OV approved by the US Food and Drug Administration (FDA), talimogene laherparepvec (T-Vec) was modified to encode exogenous GM-CSF for immune activation another HSV-1 mutant [41].Combination therapy of T-Vec and other therapeutics is currently under intensive clinical evaluations [40]. Another HSV mutant HF10 has also been clinically evaluated in several tumors. A phase I/II clinical trial of HF10 for treatment of patients with solid cutaneous tumors was completed (NCT01017185). Recently, the first intravenous administration of HSV was reported in pediatric patients with cancer, systemic delivery of this HSV mutant called HSV1716 showed good tolerability and produced a similar clinical response comparing to intratumoral HSV1716 [43, 44].

### Adenovirus

Adenovirus is a non-enveloped double-stranded DNA virus with a linear genome of 30–38 kb. Considering the well-understood genome structure and life cycle, and the stable physiological properties, genetic manipulation of an adenovirus can be easily achieved [45]. The majority of genetically engineered oncolytic adenoviruses (OAds) are derived from Ad serotype 5, which have been investigated extensively. H101 is the progeny product of ONYX-015, which is the first tumor-specific OAd mutant evaluated in clinic [42]. In 2005, it was approved by the Chinese FDA for the treatment of nasopharyngeal carcinoma in combination with cisplatin or 5-fluorouracil (5-FU), or both [46]. Other OAds that are undergoing clinical evaluations include LOAd703, VCN-01, Telomelysin (OBP-301) and ONCOS-102, clinical responses showed that these OAd recombinants produced potent anti-tumor efficacy and were well-tolerated in patients [47–51].

### Vaccinia virus (VV)

Vaccinia virus (VV) is a double-stranded DNA virus with a 180–200 kb genome. The replication, cytotoxicity, and transgene capacity of Lister strain VV is not affected by the hypoxic environment [52]. Its mutant pexastimogene devacirepvec (Pexa-Vec, also known as JX-594) with a thymidine kinase gene mutation and GM-CSF gene insertion is currently under evaluation in a phase III trial of hepatocellular carcinoma (NCT02562755). Another mutant, GL-ONC1 carrying three exogenous genes (green fluorescent protein fusion, β-galactosidase, and β-glucuronidase), is also under clinical evaluation [53, 54]

### Reovirus

This non-enveloped RNA virus contains 9–12 segments of linear double-stranded RNA. It has been reported that reovirus maintains its replicating and oncolytic abilities in the hypoxic TME, and downregulation of hypoxia inducible factor 1α (HIF-1α) has been observed during reovirus infection [55]. Moreover, through intravenous injection, reovirus can use peripheral blood mononuclear cells (PBMCs) and dendritic cells (DCs) as a cell-based carrier to evade neutralizing antibodies (NAbs) which mediate potent viral clearance, this biological property of reovirus renders a more efficient viral replication through systemic delivery [56, 57].

Reolysin (also known as pelareorep), a naturally occurring reovirus type 3 Dearing strain, has demonstrated a robust cytotoxic effect in KRAS-activated malignancies [58, 59]. Its combinations with chemotherapy, radiotherapy and immunotherapy have been evaluated in various clinical trials [60].

### Measles virus (MV)

MV is a single-stranded RNA virus. CD46 is the receptor that mediates cellular entry of MV, which is often overexpressed on tumor cells. This confers tumor selectivity to MV [61]. MV-NIS is an MV mutant that expresses sodium iodide symporter. Therefore, uptake of I^131^ by infected tumor cells is increased, which generates radiotoxicity in tumor cells [62, 63]. The safety and efficient viral replication of MV-NIS have been documented in a phase I trial of patients with recurrent multiple myeloma [64].

### Coxsackievirus A21 (CVA21 or CAVATAK)

CVA21 is a naturally occurring enterovirus that specifically targets susceptible tumor cells expressing CVA21 cellular receptors, intercellular adhesion molecule-1, and decay-accelerating factor [65]. A phase I clinical trial of CVA21 for treatment of bladder cancer has been reported [66].

There are some other OVs like Newcastle disease virus (NDV), parvovirus and myxoma virus, have been studied for treatment of various tumors [67–70]. For example, clinical evaluations of parvovirus H-1 (H-1PV or ParvOryx), a naturally occurring parvovirus strain, have undergone in patients with glioblastoma and pancreatic cancer [67, 68].

## OVs reverse immunosuppression by modulating TME components

**Fig. 2:**
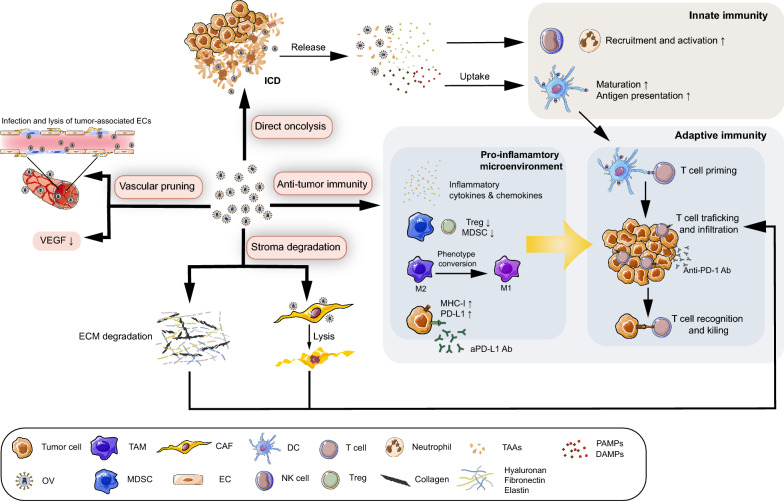
Mechanism of oncolytic viruses (OVs) targeting the TME. (i) Direct oncolysis: immunogenic cell death (ICD) induced by OVs leads to the release of numerous molecules, including pathogen-associated molecular pattern molecules (PAMPs), damage-associated molecular pattern molecules (DAMPs), and tumor-associated antigens (TAAs), which enhance activation of antigen presenting cells (APCs) such as dendritic cells (DCs). Simultaneously, infected tumor cells also produce various inflammatory cytokines such as type I interferon (IFN) and chemokines. (ii) Anti-tumor immunity: Inflammatory cytokines and chemokines are produced under OV infection, leading to the recruitment of innate immune cells such as neutrophils and natural killer (NK) cells. Antigen-loaded DCs after OV infection trigger T cell priming and degraded extracellular matrix (ECM) by OVs enhances intratumoral infiltration of T cells. The proinflammatory microenvironment created by OVs includes M2-to-M1 transition of tumor-associated macrophages (TAMs), decreased level of regulatory-T cells (Tregs) and myeloid-derived suppressor cells (MDSCs), and upregulated major histocompatibility complex-I (MHC-I) on tumor cells, which facilitate T cells to overcome immune suppression and complete the final recognition and killing step. Further immunostimulatory effect of OVs was achieved by synergizing with immune checkpoint blockade (ICB) therapy. (iii) Vascular pruning: OVs exert anti-angiogenic effects through direct lysis of tumor-associated endothelial cells (ECs) and reducing the level of vascular endothelial growth factor (VEGF), preventing the immunosuppressive effect from those angiogenic components. (iv) Stroma degradation: various ECM-degrading agents expressed by engineered OVs induce stroma degradation. Concurrently, OV-induced CAF lysis also inhibits excessive ECM production. Alleviated stroma fibrosis subsequently promotes the infiltration of T cells

Generally, the multifaceted roles of OVs in tumors are performed through several mechanisms [71] as is shown in Fig. 2. (1) Direct oncolysis: In the context of viral infection, OVs selectively infect and replicate in tumor cells, leading to lysis of tumor cells and subsequent release of viral progeny and tumor cell components. (2) Anti-tumor immunity: Followed by OV-induced immunogenic cell lysis, pathogen-associated molecular pattern molecules (PAMPs), damage-associated molecular pattern molecules (DAMPs), and tumor-associated antigens (TAAs) are released, triggering rapid but unspecific innate immune responses. These reactions enhance tumor and viral antigen presentation by DCs, leading to subsequent T cell priming and activation, and ultimately creating an immunostimulatory microenvironment [72]. By taking advantage of this immune-potentiating ability, enhanced anti-tumor efficacy of OVs is achieved. (3) Vascular pruning: Studies have shown that OVs act as an anti-angiogenic agent by directly targeting tumor-associated ECs and proangiogenic factors through stimulating host immune cells to produce anti-angiogenic factors to reshape the tumor vasculature [4]. (4) Stroma degradation: OVs attract neutrophils that are a potent ECM modifier to decompose the ECM [73]. Additionally, stroma-decomposing agents have been integrated into OV genomes to alleviate fibrotic reactions and facilitate drug spreading and immune cell infiltration within tumors.

Next, we focus on the mechanisms and applications of oncolytic immunotherapy in reversing the immunosuppressive TME during the anti-tumor immune cycle.

### Activation of innate immunity under OV infection

Soon after viral infection and subsequent oncolysis, non-specific innate immunity is triggered rapidly. Cytokines, such as type-I IFN and tumor necrosis factor-α (TNF-α), are secreted by infected tumor cells, TAAs and viral PAMPs are exposed to the host immune system followed by oncolysis [74]. This virus-induced cell lysis is recognized as immunogenic cell death (ICD) characterized by the release of DMAPs such as ATP, high-mobility group box 1, and calreticulin. DCs are then recruited and convert to the mature phenotype under exposure to DAMPs and TAAs [75, 76]. Toll-like receptors on immune cells are activated by DAMPs and PAMPs, leading to subsequent release of proinflammatory cytokines [e.g. tissue necrosis factor-α (TNF-α), type-I IFN, IL-1β, IL-6, and IL-12] and chemokines, resulting in recruitment and activation of innate immune cells such as neutrophils and NK cells [77, 78].

### OV infection facilitates adaptive immune responses

T cell-mediated tumor-specific immune responses are the mainstay of adaptive immunity during OV infection. It has been demonstrated that depletion of T cells leads to absent anti-tumor efficacy despite observing persistent OV replication [79]. However, tumor-infiltrated lymphocytes (TILs) are extensively suppressed by TME components. Therefore, the final goal of oncolytic immunotherapy is to assist T cells in overcoming immunosuppressive barriers and become fully activated to exert tumoricidal activity. According to Twumasi-Boateng et al., both armed and unarmed OVs participate in the whole process of T cell activation, including priming, trafficking, infiltration, activation, and final tumor killing [80].

First, after OV-triggered innate immune responses, antigen-loaded APCs migrate to draining lymph nodes and initiate T cell priming by presenting antigens to naive T cells. Subsequently, production of lymphocyte-recruiting chemokines and proinflammatory cytokines during the virus-induced type-I IFN response leads to the recruitment and activation of T cells. Secreted inflammatory factors such as TNF-α during OV infection also upregulate the expression of selectin in ECs, allowing enhanced extravasation of lymphocytes from the vasculature, which is an EC-dependent process [80–82]. Then, during T cell infiltration, OVs alleviate structural barriers in the fibrotic tumor stroma by targeting stromal components and attracting neutrophils that secrete proteases such as matrix metalloproteinases (MMPs) and elastase to degrade the ECM [73, 83]. Increased infiltration of CTLs under OV infection has been observed in both preclinical and clinical studies, which is related to a better prognosis [84–87]. Finally, during conjugation and the killing step, OVs reverse the immunosuppressive phenotype of immunoinhibitory cells and upregulate MHC-I on the tumor cell surface, so that T cells are able to escape from immunosuppression and achieve efficient tumor recognition and killing [88–90].

Apart from localized immune responses, local administration of many OV agents also induces systemic immune responses as demonstrated by tumor regression, increased accumulation of immune cells, and upregulated immune-related gene expression in non-injected sites, which is referred as the “abscopal effect” [91–93]. Collectively, this series of immune responses caused by viral infection ultimately induces potent and durable anti-tumor immunity [91, 92].

Despite the immunomodulatory effect of OVs, the immune system acts as an opposing force against OVs. Such obstacles are extremely intractable when OVs are injected intravenously. Virus opsonization mediated by neutralizing antibodies (NAbs), complement proteins, erythrocytes, and other blood components, and the following virus phagocytosis in the liver and spleen impair the effectiveness of OVs to a great extent [94]. Therefore, approaches to induce fully engaged anti-tumor immunity by OVs while minimizing immune-mediated viral clearance require further exploration.

## Genetically engineered OVs as a promising immunotherapeutic agent

To further potentiate the anti-tumor immunity of OVs, many genetically engineered OV mutants armed with various therapeutic genes have been investigated intensively.

### Expression of immunostimulatory molecules by OVs

To restore the impaired function of APCs in the immunosuppressive TME, OVs have been used as an enhanced cancer vaccine by inserting TAAs into the viral genome to improve T cell priming [95, 96]. Additionally, treatment with OVs armed with proinflammatory cytokines such as GM-CSF, interleukins, and TNF-α, is a common strategy. The feasibility of GM-CSF insertion was confirmed by successful clinical translation of T-VEC [41], and promising clinical outcomes of other types of GM-CSF-inserted OV recombinants are anticipated, such as ONCOS-102 and Pexa-Vec. Genetic insertion of interleukins, such as IL-2, IL-7, and IL-12, has also been applied to enhance activation of lymphocytes and NK cells [97, 98]. For example, synergistic activation of T cells by the combination of IL-12 and IL-7 in vitro has been reported [99]. Consequently, a recent study designed a novel VV recombinant encoding both IL-7 and IL-12. Compared with IL-12 or IL-7 alone, coexpression of IL-12 and IL-7 by the VV caused a significant increase in activated TILs characterized by increased expression of granzyme B and genes related to T cell functions. Additionally, this combination increased tumor susceptibility to ICB treatment [97]. Apart from cytokines, chemokines are also ideal factors to enhance recruitment of immune cells. A recent study enhanced adoptive NK cell transfer therapy by strengthening the CCR5-CCL5 axis that is crucial for NK cell migration to tumors. By delivering CCL5 via a CCL5-encoding lentivirus, recruitment and intratumoral accumulation of modified NK cells overexpressing CCR5 was enhanced significantly. The combination group achieved a superior prognosis with a complete response rate of > 50% in mice [100]. In some cases, cytokines and chemokines have been concomitantly incorporated into OVs for better immunostimulatory effects [101, 102].

### Expression of ICB proteins by OVs

Emerging applications of ICB have broadened the application strategies of oncolytic immunotherapy. Because toxicities related to systemic administration of checkpoint inhibitors have been reported in the clinic [103], OVs provide a rational delivery approach by locally expressing checkpoint inhibitors in tumor regions. Recently, a vaccinia virus coexpressing a PD-L1 inhibitor and GM-CSF (VV-iPDL1/GM) was generated and exerted potent systemic anti-tumor effects in several preclinical tumor models. Blockade of PD-L1 expression in both tumor and immune cells, enhanced neoantigen presentation on tumor cells, and improved infiltration and activation of T cells were achieved by VV-iPDL1/GM [104]. Similarly, a myxoma virus mutant expressing a soluble form of PD1 (vPD1) was evaluated in an immunocompetent murine model of B16-F10 melanoma, which showed poor immunogenicity. Compared with the combination of anti-PD1 monoclonal antibodies and the unmodified myxoma virus, vPD1 monotherapy led to more efficient anti-tumor responses and significant survival benefits [105]. Another study using the B16-F10 melanoma mouse model reported that NDV together with radiotherapy-induced significant tumor regression in ICB-treated tumors compared with single treatment. Additionally, an NDV encoding an anti-CTLA4 single-chain variable fragment was created to combine with radiotherapy. The survival outcome was comparable to the combination of radiotherapy and anti-CTLA4 mAbs, which indicated a radiosensitizing effect of NDV and potential in combination with radiotherapy and ICB therapy [106].

### Expression of bispecific T-cell engagers by OVs

OVs have also been designed to express bispecific T-cell engagers (BiTEs), which simultaneously bind to TAAs on tumor cells and CD3 on T cells, so that infiltrated T cells are retargeted to tumor cells. For hematopoietic malignancies, BiTEs have progressed to clinical applications. As an example, Blinatumomab is a CD19/CD3 BiTE that has been clinically approved for treating B-cell precursor acute lymphoblastic leukemia [107]. However, BiTEs hardly penetrate into solid tumors because of structural barriers and poor tumor perfusion. Therefore, using OVs as a carrier to achieve local BiTE expression has overcome this physical barrier to a great extent. For example, an MV mutant was generated to deliver BiTEs that target CD3 and CD20 or CEA (VV-BiTE). Durable anti-tumor immunity was achieved through increased infiltration and cytotoxicity of CTLs. Moreover, synergistic effects and prolonged survival were observed in patient-derived colon cancer spheroids when VV-BiTE was combined with human peripheral blood monocytes (PBMCs) [108]. BiTE-armed OVs can also target both tumor and stromal cells such as CAFs. Fibroblast activation protein-α (FAP-α) is overexpressed on CAFs. Therefore, FAP-α-targeted BiTEs (FAP-BiTEs) that mediate binding between T cells and FAP-expressing CAFs were genetically introduced into OAds. In addition to direct oncolysis, T cell activation, cytotoxicity against CAFs, and diminished CAF-associated immunosuppressive factors were achieved by OAds expressing FAP-BiTEs [109, 110]. OVs expressing BiTEs have also been applied to facilitate CAR-T cell therapy [111], which will be introduced later.

### Expression of stromal regulators by OVs

To improve intratumoral infiltration of immune cells and the OV itself, strategies of manipulating OVs to alleviate physical barriers in the TME have been considered. As the critical driver of the fibrotic network in the TME, CAFs are considered a common therapeutic target. Apart from the aforementioned FAP-BiTEs expressed by OVs. Urokinase receptor (uPAR) has also been considered as a target because of its overexpression on both tumor and stromal cells. Therefore, a uPAR-retargeted MV has been designed. Virus-mediated regulation of tumor-stroma interactions, including decreased ECs and fibroblasts, and downregulated gene expression associated with stromal components and angiogenesis, have been observed in several tumor models [112, 113]. In terms of targeting the ECM, hyaluronan is a critical ECM component responsible for tissue elasticity and water retention, which is closely related to elevated IFP in tumor stroma [114]. By integrating hyaluronidase into an OV genome to decompose overexpressed hyaluronan, physical barriers of the TME have been alleviated [115–117]. Apart from hyaluronidase, MMPs have also been expressed by OVs to enzymatically degrade ECM proteins such as collagen [118, 119]. Tumor-derived TGF-β is a critical signaling molecule that plays essential roles in the phenotypic conversion of CAFs and immunosuppression [120]. OVs have been designed to directly target TGF-β by expressing TGF-β-antagonizing molecules such as decorin [121]. The practicality of this method is supported by solid preclinical evidence including ECM degradation, immune activation, improved intratumoral spreading within tumors, and anti-tumor efficacy of OVs [122–125].

## OVs in combination with immunotherapy

The proinflammatory TME created by OVs renders a tumor more susceptible to immunotherapy. Effective tumor eradication has been achieved in both preclinical and clinical studies that combined OVT with immunotherapy. Next, the applications of OVs in combination with immunotherapy will be introduced, as is shown in Table 2.

**Table 2:** Clinical trials of OVs in combination with immunotherapy

Combination strategy	OV type	OV mutant	Combination agent	Targeted cancer	Trial phase	Trial status	Trial No.
Immunotherapy	HSV-1	T-Vec	Pembrolizumab (anti-PD1)	Melanoma	II, III	Active (not recruiting)	NCT02263508, NCT02965716
			Pembrolizumab	Head and neck cancer	I	Active (not recruiting)	NCT02626000
			Ipilimumab (anti-CTLA4)	Sarcoma	II	Recruiting	NCT03069378
			Ipilimumab	Melanoma	I/II	Active (not recruiting)	NCT01740297
			Ipilimumab + Nivolumab (anti-PD1)	Breast cancer	I	Recruiting	NCT04185311
			Atezolizumab (anti-PD-L1)	Breast cancer, colorectal cancer	I	Recruiting	NCT03256344, NCT03802604
		HF10	Ipilimumab	Melanoma	II	Completed	NCT02272855
			Nivolumab	Melanoma	II	Active (not recruiting)	NCT03259425
	Adenovirus	LOAd703	Atezolizumab	Melanoma	I/II	Recruiting	NCT04123470
		VCN-01	Durvalumab (anti-PD-L1)	Head and neck cancer	I	Recruiting	NCT03799744
		Telomelysin	Pembrolizumab	Advanced solid tumors	I	Recruiting	NCT03172819
		ONCOS-102	Durvalumab	Peritoneal malignancies	I/II	Recruiting	NCT02963831
	Reovirus	Reolysin®	Pembrolizumab	Pancreatic cancer	II	Active (not recruiting)	NCT03723915
			Nivolumab	Multiple myeloma	I	Recruiting	NCT03605719
	Vaccinia virus Vaccinia virus	Pexa-Vec Pexa-Vec	Ipilimumab	Advanced solid tumors	I	Recruiting	NCT02977156
Immunotherapy			Durvalumab + Tremelimumab (anti-PD-L1)	Colorectal cancer	I/II	Recruiting	NCT03206073
			Nivolumab	Hepatocellular carcinoma	I/II	Active (not recruiting)	NCT03071094
	Coxsackie virus	CAVATAK	Pembrolizumab	Melanoma	I	Completed	NCT02565992
			Pembrolizumab	Non-small cell lung carcinoma, bladder cancer	I	Completed	NCT02043665
			Ipilimumab	Melanoma	I	Completed	NCT02307149, NCT03408587
			Pembrolizumab	Non-small cell lung carcinoma	I	Active (not recruiting)	NCT02824965
Multi-therapy	Adenovirus	LOAd703	Atezolizumab + Gemcitabine + Nab-paclitaxel	Pancreatic cancer	I/II	Recruiting	NCT02705196
		ONCOS-102	Pembrolizumab + Cyclophosphamide	Melanoma	I	Active (not recruiting)	NCT03003676
	Reovirus	Reolysin®	Pembrolizumab + Gemcitabine + FOLFIRI	Pancreatic cancer	I	Completed	NCT02620423

### Combining OVs with ICB therapy

Immunotherapeutic effects of ICB therapy act in a T cell-dependent manner, while the absence of CTLs is an intractable problem in many tumors with low immunogenicity, which leads to a limited response rate [126, 127]. Both preclinical and clinical studies have revealed that expression of ICB proteins during OV infection, such as PD-L1 and PD-1, was upregulated in both tumor cells and immune cells, and increased T cell trafficking and infiltration into the tumor were observed [85, 128]. Blockade of PD1/PD-L1 and CTLA4 pathways by ICB therapy further enhance activation of recruited CTLs, thereby achieving a synergistic effect.

In an ex vivo melanoma coculture model, upregulated ICB protein, DC maturation, and a decreased TGF-β level were observed after H-1PV infection, and addition of ipilimumab or nivolumab further augmented this immunostimulatory effect characterized by further reduction of the TGF-β level, an increased number of granzyme B^+^ CD8^+^ T cells, and increased release of granzyme B, IFNγ, and TNFα [129]. This synergistic effect was further supported by intensive in vivo studies. In an ovarian cancer mouse model, a tumor antigen-armed Maraba virus increased intratumoral CD8^+^ T cell infiltration, and local T cell suppression via PD-1/PD-L1 axis was subsequently alleviated by anti-PD-1 therapy. Suppressed tumor progression was also achieved by combination therapy [95]. Consistent results were also obtained in a triple-negative breast cancer model using Maraba virus as a neoadjuvant therapy before ICB therapy [130]. Similarly, a study combined VV and PD-L1 blockade in a colon cancer model. Immunological results showed that OV-induced PD-L1^+^ cells and immunosuppressive cells, such as TAMs, MDSCs, and Tregs, were significantly reduced in the combination group and influx of CTLs with restored cytotoxicity was increased. These synergistic effects collectively alleviated the tumor burden and improved the survival outcome [131]. Inhibition of the leading immunosuppressive factor TFG-β by OVs has also shown synergistic efficacy with ICB therapy. Xu et al., recently designed an OAd encoding a soluble TGFβ receptor II fused with a human IgG Fc fragment (sTGFβRIIFc), which is a TGFβ decoy that inhibits TGFβ signaling. In immunocompetent mouse models of breast and renal cancers, addition of the sTGFβRIIFc-expressing OAd significantly augmented anti-PD-1 and anti-CTLA-4-mediated inhibition of tumor metastasis [132]. In addition to the aforementioned ICB proteins, other ICB proteins are also considered as therapeutic targets. T cell immunoglobulin domain and mucin domain-3 (TIM3) is an ICB protein expressed on immune cells, which correlates with resistance to immunotherapy [133]. A recent study revealed that OVT together with PD-1 blockade was unable to improve T cell activation in a lung cancer mouse model, whereas dual blockade of PD-1 and TIM3 enhanced the viro-immunotherapy. Upregulation of TIM3 on CD8^+^ T cells and TIM3 ligand on tumor cells induced by OVs contributed to the resistance to PD-1 blockade. This study suggested that targeting multiple ICB pathways is a rational approach to overcome resistance to single ICB treatment and achieve optimal synergy with OVT [134].

With the prevalence of “cocktail” therapy, adding other immunostimulatory agents to this combination regimen has been considered because it minimizes dose-dependent toxicity and drug resistance. For example, colony-stimulating factor 1 (CSF-1) is crucial for the recruitment and differentiation of TAMs, which is highly expressed in tumors. A study reported that combining OAds with anti-PD-1 therapy plus a CSF-1 receptor inhibitor greatly enhanced tumor regression and overall survival, which were achieved through increased functional T cell infiltration and anti-tumor phenotype conversion of TAMs [135]. Additionally, enhanced viral replication, T cell activation, and anti-tumor efficacy in mice with melanoma were achieved by triple combination of T-Vec, anti-PD-1 therapy, and MEK inhibition that targets a BRAF mutation [136].

OV-induced potentiation of ICB therapy has been strongly supported by substantial clinical evidence. In a window-of-opportunity clinical study, nine participants with high-grade glioma were recruited and intravenously administrated with Reolysin. Tumor samples from virus-treated patients showed efficient viral replication and increased CTL infiltration as well as more intensive PD-1 and PD-L1 expression, which were mediated by IFNs. Sequential treatment with Reolysin® prior to PD-1 blockade in a preclinical glioma model led to further immune activation and survival benefits [137]. Notably, a phase Ib clinical study conducted by Ribas et al., reported that in patients with advanced melanoma, the intra-tumoral PD-L1 expression was upregulated after T-Vec injection. Combining T-Vec with anti-PD-1 antibody achieved a high overall response rate (ORR) of 62%. Increased CD8^+^ T cells infiltration and IFN gene expression in post-treatment tumor samples explained this synergistic efficacy, providing a solid evidence of oncolytic immunotherapy [85]. Subsequently, a phase II clinical trial that combined T-Vec with pembrolizumab (anti-PD-1 mAb) reported an optimal ORR of 30% in 20 patients with advanced sarcoma. Immunological data from patients also confirmed the augmented anti-tumor immunity [138]. Similarly, 198 patients with advanced melanoma were recruited in another phase II trial and the combination of T-Vec and ipilimumab (anti-CTLA4 mAb) showed a superior ORR than ipilimumab alone (39% vs. 18%) [139].

Triple combination of chemotherapy, ICB therapy, and OVT has been applied in the clinic because synergistic efficacy has been reported between OVs and some chemical agents such as gemcitabine and paclitaxel [140, 141]. Recently, this triple combination strategy was applied in a phase Ib trial treating patients with unresectable PDAC. Combination of Reolysin, pembrolizumab, and first-line chemotherapy regimen (gemcitabine, irinotecan, leucovorin, and 5-FU) of PDAC achieved a disease control rate of 27%, and immunological data were highlighted in this study. High clonality and expansion of peripheral T cells and increased expression of inflammatory genes indicated an immune activation in patients with clinical benefits [142].

Currently, extensive clinical trials combining OVs with ICB therapy are under evaluation and promising outcomes are anticipated.

### Combining OVs with CAR-T cell therapy

The existence of the TME in solid tumors poses intractable challenges for CAR-T cell therapy, including inadequate CAR-T cell infiltration caused by fibrotic stroma and suppressed CAR-T cell functions due to the immunosuppressive components. To facilitate the recruitment of CAR-T cells, a VV loaded with CXCL11 was designed. Two delivery approaches of CXCL11 were established. CXCL11 was expressed either via CAR T cells (CAR/CXCL11) or vaccinia virus (VV.CXCL11). Compared with CAR/CXCL11, VV.CXCL11 significantly increased intratumoral accumulation of CAR-T cells and potent anti-tumor efficacy was observed, which indicated the therapeutic potential of OVs in assisting CAR T cell therapy [143]. Another study combined CAR-T cells targeting folate receptor alpha with an OAd expressing an epidermal growth factor receptor-targeting BiTE. This combination induced robust T cell activation and proliferation both in vitro and in vivo with improved tumoricidal efficacy and survival compared with single agent treatment [111].

CAR-T cell therapy can be further facilitated by the combination of OVs and ICB therapy. As mentioned previously, local expression of checkpoint inhibitors by OVs alleviate the adverse effects caused by systemic delivery of checkpoint antibodies. Therefore, an OAd encoding both a PD-L1-blocking antibody and IL-12p70 (CAd12_PDL1) was designed to combine with HER2-specific CAR T cell therapy. In a xenograft model of head and neck cancer, compared with the insufficient anti-tumor efficacy of single therapy, combining systemic delivery of CAR T cells and local treatment of CAd12_PDL1 significantly improved the survival outcome. Control of tumor growth in both primary and metastatic sites was also achieved by the combination therapy in the orthotopic model [144]. Consistent results were also reported by Tanoue et al. Compared with the combination of anti-PD-L1 IgG and CAR T-cells, the addition of a OAd expressing anti-PD-L1 mini-antibody had superior therapeutic efficacy in a xenograft model of HER2^+^ prostate cancer [145].

To date, no clinical investigation of OVs in combination with CAR-T cell therapy has been reported.

## Current challenges and future directions

The development of OVT has led to anti-tumor therapy beyond the traditional concept. Although promising results have been obtained by employing OVs in TME-targeted therapy, a multitude of challenges remain to be overcome.

### Optimizing preclinical models to simulate the human TME

The abundant and sophisticated components in the TME render traditional preclinical models such as cell-derived tumor models insufficient to gain insights into the TME. Moreover, despite animal models, such as murine and canine models, sharing similarity with humans, species variation is hard to overcome, resulting in discrepant results between preclinical models and clinical patients. Although the patient-derived xenograft (PDX) model has achieved better translation with its well-preserved biological properties, such as mutation profiles and drug sensitivity of the primary tumor and its surroundings [146], the immunocompromised mice used for PDX engraftment are not suitable to evaluate oncolytic immunotherapy [147]. Hence, a humanized mouse model with a human immune system was established by injecting human leucocyte antigen (HLA)-matched PBMCs or cord blood CD34^+^ hematopoietic stem cells into an immunocompromised mouse. Thus far, the immunomodulatory effect of OVs has been evaluated in several studies using humanized mice [97, 148, 149]. Furthermore, by joining PDX and a humanized mouse model together, a novel preclinical model termed as Hu-PDX with human tumor cells, stroma, and an immune system is considered as the most desirable tool for drug screening or as an “avatar” model to predict clinical responses of patients. Several studies evaluating immunotherapy, especially ICB therapy, in solid tumors using a Hu-PDX model have been reported [150–152]. It is conceivable that introducing OVT into Hu-PDX models together with immunotherapy provides a great insight into translational research and precision medicine.

So far, developing Hu-PDX models to investigate the interactions between OVs and host immunity together with immunotherapy still largely lacks in-depth research. Limitations in performing such studies are as follows. (1) The acquirement of a PDX is difficult considering that many patients are diagnosed at an advanced stage with no indication for surgery. (2) The establishment of a Hu-PDX model is time consuming and costly with strict requirements of bringing two sophisticated biological systems together, such as HLA matching and stable engraftment. Therefore, technical progress in this field is urgently needed.

### Minimizing immune-mediated viral clearance to achieve systemic delivery of OVs

The therapeutic benefits and safety of OVs have been demonstrated using IT administration, While some additional benefits, such as simplified operation, reaching metastatic sites, and easily triggered systemic immune responses, can be achieved by IV administration [153]. However, in practice, several challenges in IV injection result in limited administration of OVs in solid tumors. Through IV injection, the concentration of OVs is diluted by the bloodstream, and rapid viral clearance is mediated by NAbs and other blood components such as complement proteins and erythrocytes, resulting in only a fraction of OVs accessing tumor sites, leading to the limited infectivity and efficacy [154, 155].

To overcome these limitations, several methods have been employed. Capsid-modified OVs have been developed to prevent OVs from virus neutralization in circulation. For example, a high level of NAbs has been a problem for highly immunogenic HSV. By genetically modifying the envelope glycoproteins on HSVs, evasion from viral neutralization has been achieved [156, 157]. Alternatively, OAds covalently coated with PEG- or poly [N-(2-hydroxypropyl) methacrylamide] (PHPMA)-based polymers have been shown to induce a low titer of Nabs, reduced anti-virus immune responses, and increased circulation time and intratumoral accumulation of OAds [158, 159].

Another promising delivery approach is cell-based carriers. MSCs are considered as the most promising OV carrier based on their inherent tumor-homing and immune-silencing properties [160, 161]. Several preclinical and clinical studies have highlighted the transportation capability of OV-loaded MSCs. For example, delivery of VVs by MSCs augmented viral amplification and delivery by alleviating anti-virus immune responses [162]. An autologous MSC carrying OAds called Celyvir has recently completed its phase I clinical trial [163]. Despite showing promise, challenges including the strict condition for ex vivo infection culture, prevention of premature carrier cell lysis, and allogenic rejections form a formidable barrier to clinical translation considering the current technical restrictions [164].

## Conclusions

OVs reverse the predicament of immunotherapy by creating a proinflammatory TME with the prevalence of “cocktail” combination therapy, and combining OVs with immunotherapy and other therapies such as chemotherapy and anti-angiogenic therapy is awaiting to be further explored. Despite some achievements, there are still many limitations in oncolytic immunotherapy. Because of the potent immune-mediated viral clearance in circulation, OVs can hardly access distant metastatic sites, which results in limited synergistic effects with immunotherapy. Methods such as cell carriers have been developed to achieve minimal anti-viral immune responses as well as optimal viral replication and spreading. Additionally, the Hu-PDX model is expected to shed light on virus-immunity interactions in the context of the humanized TME, which is important to achieve successful clinical translation. Collectively, oncolytic immunotherapy has built a solid basis for treating malignancies, a new era of anti-cancer therapy on the basis of oncolytic virotherapy is anticipated.

## Data Availability

Not applicable.

## Abbreviations

APC: Antigen presenting cell
BiTE: Bispecific T-cell engager
CAF: Cancer-associated fibroblast
CCL: Chemoattractant cytokine ligand
CRC: Colorectal carcinoma
CTLA4: Cytotoxic T lymphocyte-associated antigen 4
DC: Dendritic cell
DAMP: Damage-associated molecular pattern molecule
EC: Endothelial cell
ECM: Extracellular matrix
FAP-α: Fibroblast activation protein-α
FDA: US Food and Drug Administration
FOLFIRI: Leucovorin and fluorouracil plus irinotecan
GM-CSF: Granulocyte–macrophage colony-stimulating factor
HLA: Human leucocyte antigen
HMGB1: High-mobility group box 1
HSV: Herpes simplex virus
H-1PV: Parvovirus H-1
ICB: Immune checkpoint blockade
ICD: Immunogenic cell death
IDO: Indoleamine-2,3-dioxygenase
IFN: Interferon
IFP: Interstitial fluid pressure
IL: Interleukin
IT: Intratumoral
IV: Intravenous
MDSC: Myeloid-derived suppressor cell
MHC-I: Major histocompatibility complex-I
MSC: Mesenchymal stem cell
MMP: Matrix metalloproteinase
MV: Measle virus
NAb: Neutralizing antibody
NDV: Newcastle disease virus
NK cell: Natural killer cell
OAd: Oncolytic adenovirus
ORR: Overall response rate
OV: Oncolytic virus
OVT: Oncolytic virotherapy
PDAC: Pancreatic ductal adenocarcinoma
PAMP: Pathogen-associated molecular pattern molecule
PBMC: Peripheral blood mononuclear cell
PDX: Patient-derived xenograft
PD-L1: Programmed cell death protein ligand-1
TAA: Tumor associated-antigen
TAM: Tumor-associated macrophage
TGF-β: Transforming growth factor-β
TIL: Tumor-infiltrated lymphocytes
TLRs: Toll-like receptors
TME: Tumor microenvironment
TNF-α: Tumor necrosis factor-α
Treg: Regulatory T cell
MSC: Mesenchymal stem cell
T-Vec: Talimogene laherparepvec
uPAR: Urokinase receptor
VEGF: Vascular endothelial growth factor
VV: Vaccinia virus
5-FU: 5-Fluorouracil
